# Rethinking HIV prevention to prepare for oral PrEP implementation for young African women

**DOI:** 10.7448/IAS.18.4.20227

**Published:** 2015-07-20

**Authors:** Connie L Celum, Sinead Delany-Moretlwe, Margaret McConnell, Heidi van Rooyen, Linda-Gail Bekker, Ann Kurth, Elizabeth Bukusi, Chris Desmond, Jennifer Morton, Jared M Baeten

**Affiliations:** 1 Department of Global Health, University of Washington Seattle, WA, USA; 2 Department of Medicine, University of Washington Seattle, WA, USA; 3 Department of Epidemiology, University of Washington Seattle, WA, USA; 4 Wits Reproductive Health and HIV Institute, University of the Witwatersrand, Johannesburg, South Africa; 5 Department of Global Health and Population, Harvard T.H. Chan School of Public Health Boston, MA, USA; 6 Human Sciences Research Council, Durban, South Africa; 7 The Desmond Tutu HIV Centre, University of Cape Town, Cape Town, South Africa; 8 College of Nursing, New York University New York, NY, USA; 9 Kenya Medical Research Institute, Nairobi, Kenya

**Keywords:** HIV, prevention, pre-exposure prophylaxis, Africa, women

## Abstract

**Introduction:**

HIV incidence remains high among young women in sub-Saharan Africa in spite of scale-up of HIV testing, behavioural interventions, antiretroviral treatment and medical male circumcision. There is a critical need to critique past approaches and learn about the most effective implementation of evidence-based HIV prevention strategies, particularly emerging interventions such as pre-exposure prophylaxis (PrEP).

**Discussion:**

Women in sub-Saharan Africa are at increased risk of HIV during adolescence and into their 20s, in part due to contextual factors including gender norms and relationship dynamics, and limited access to reproductive and sexual health services. We reviewed behavioural, behavioural economic and biomedical approaches to HIV prevention for young African women, with a particular focus on the barriers, opportunities and implications for implementing PrEP in this group. Behavioural interventions have had limited impact in part due to not effectively addressing the context, broader sexual norms and expectations, and structural factors that increase risk and vulnerability. Of biomedical HIV prevention strategies that have been tested, daily oral PrEP has the greatest evidence for protection, although adherence was low in two placebo-controlled trials in young African women. Given high efficacy and effectiveness in other populations, demonstration projects of open-label PrEP in young African women are needed to determine the most effective delivery models and whether women at substantial risk are motivated and able to use oral PrEP with sufficient adherence to achieve HIV prevention benefits.

**Conclusions:**

Social marketing, adherence support and behavioural economic interventions should be evaluated as part of PrEP demonstration projects among young African women in terms of their effectiveness in increasing demand and optimizing uptake and effective use of PrEP. Lessons learned through evaluations of implementation strategies for delivering oral PrEP, a first-generation biomedical HIV prevention product, will inform development of new and less user-dependent PrEP formulations and delivery of an expanding choice of prevention options in HIV prevention programmes for young African women.

## Introduction: HIV epidemiology and prevention in young women in Africa

Young women aged below 25 account for three of the almost four million young people in sub-Saharan Africa who are living with HIV and have one of the highest HIV incidence rates globally [1]. New prevention tools including community-wide HIV testing, antiretroviral treatment (ART) as prevention, and voluntary medical male circumcision have shown tremendous promise and implementation is progressing. However, women, particularly young women, have not maximally benefited from these interventions because they are not directly under women's control. Women are also are at increased risk because their male sexual partners are less likely to be HIV tested and know their HIV serostatus [2], and their HIV infected partners are less likely to have viral suppression due to late ART initiation and higher drop-off in the HIV care continuum [3].

Women are particularly vulnerable to HIV during adolescence and their early 20s due to biological, behavioural and structural factors. Biological factors that increase HIV susceptibility among women include co-infection with sexually transmitted infections (STIs) [4–7], genital inflammation [8], and their male partners’ circumcision status [9, 10] and viral load [11, 12]. Hormonal influences such as pregnancy [13] and injectable contraception [14] have also been shown in observational studies to increase HIV risk, although the data are not conclusive. Individual-level behavioural factors that contribute to the increased vulnerability of young African women to HIV infection include younger age at sexual debut [15], older sexual partners [16, 17], concurrent sexual partners [18, 19], lack of consistent condom use [20], interpersonal physical and sexual violence [21, 22], and alcohol abuse [23]. Contextual factors such as poverty and the low social power of young people in many societies also frame and drive sexual decision-making and risk [24]. Gender inequities, through norms and unequal gender power relations, also play an important role in determining young women's risk for intimate partner violence (IPV) and vulnerability in contracting HIV [25].

HIV incidence remains high among young African women, and few strategies have demonstrated effectiveness in reducing new HIV infections in this population. Until recently, the mainstay of HIV prevention for young men and women was behaviour change focused on delay of sexual debut, decreasing the number of sexual partners and increasing condom use through interventions delivered to individuals, couples, families, peer groups or networks, institutions or entire communities [26, 27]. Several major behavioural interventions focused on African youth had a school-based component [28–33], which provided easy access to young people, but were challenged by conservative teachers and parents uncomfortable with teaching sexual health topics to students [27]. Many youth-based interventions were focused on providing information about safe sex and teaching skills about condom negotiation but did not attempt to influence social factors or broader structural factors that shape sexual behaviour [34]; these social and structural factors are difficult to address, especially in the context of any single research study. For many women, sexual relationships involve tacit understandings where they receive consumer items, monetary gifts or other benefits and necessities from their sexual partner, and may not be able to negotiate condom use [35–40]. Encouraging results have been observed with interventions that focused on gender norms and equity in South Africa, which have shown reductions in IPV, improvements in women's well-being in the IMAGE study [41] and modest reductions in sexual risk and HSV-2 incidence in the Stepping Stones project [31]. A community-level intervention to change attitudes, social norms and behaviours related to IPV, HIV disclosure and risk reduction in women seeking HIV testing in Uganda demonstrated a reduction in some forms of IPV against women and a reduction in HIV incidence [42].

There is an urgent need for effective prevention for young African women given annual HIV incidence rates of 5–6% and higher in some settings. Oral antiretroviral pre-exposure prophylaxis (PrEP) is currently the prevention intervention for which the evidence for efficacy is greatest, if uptake and adherence are high, as described elsewhere in this issue and briefly summarized below. PrEP also is a strategy that can be independent of the partner's knowledge or “buy-in,” which could be relevant for young women who have a history of IPV or fear their partner's reaction to their efforts to reduce their risk of HIV. We focus on the barriers, opportunities and implications for implementing PrEP in young African women by reviewing behavioural and behavioural economic approaches to HIV prevention for this group. Although efforts to identify more effective and scalable behavioural and structural interventions and longer-acting, less user-dependent PrEP formulations for women must continue, it is important to evaluate the potential for PrEP as a core component of combination prevention for this key population that also includes behavioural and structural interventions, as recommended by the US President's Emergency Plan for AIDS Relief initiative for adolescent girls and young women: Determined, Resilient, Empowered, AIDS-free, Mentored and Safe (DREAMS) [43]. Defining the proportion and characteristics of women who are likely to use oral PrEP and evaluating scalable adherence strategies and delivery models will be informative in understanding delivery of long-acting PrEP formulations, multiprevention technologies and other prevention modalities to this key population.

## Discussion: evidence for PrEP as a primary HIV prevention strategy

PrEP with oral tenofovir (TDF) or TDF co-formulated with emtricitabine (TDF/FTC or Truvada^®^) demonstrated substantial HIV prevention benefits (up to 75% reduction in HIV incidence) in four trials conducted among men who have sex with men (MSM) in a multicountry trial, injection drug users in Asia, and African HIV serodiscordant couples and young men and women [44–48]. Subgroup analyses estimated efficacy to be very high (range from 80 to 92%) among those who had tenofovir in their blood samples [44–47]. PrEP efficacy among women was high (approximately 70% compared to placebo and approximately 90% when detected in blood) in the Partners PrEP Study in all women and in key subgroups, including younger women [49, 50]. In contrast, in the VOICE and FEM-PrEP trials, adherence to PrEP was very low (<30% based on a random subset with drug levels) and no efficacy was observed [51, 52]. Pharmacokinetic studies have indicated significantly lower concentrations of tenofovir in vaginal than rectal tissues [53, 54], suggesting PrEP may be less forgiving to adherence for women compared with MSM. Pharmacometric modelling is being conducted to better understand the relationship of PrEP drug exposure to efficacy by gender [55]. In addition to drug exposure, other variables, such as concomitant STIs and genital inflammation that increase HIV susceptibility [8], could modulate the efficacy of PrEP. However, the overwhelming evidence is that PrEP is biologically effective in both men and women, when taken.

The results of oral PrEP in the VOICE and FEM-PrEP trials in young African women have prompted a range of questions about next steps in HIV prevention: Did low PrEP adherence reflect low HIV risk perception; lack of motivation in the clinical trial context where they could receive placebo or an unproven product; lack of self-efficacy, external factors that limited prevention uptake, stigma, an inability to take a daily pill; a lack of motivation and interest in HIV prevention, in general, or competing daily priorities where trade-offs between current benefits and future prevention are made? Qualitative data from VOICE indicate that factors that contributed to low uptake and adherence included uncertainty and ambivalence about using antiretrovirals for prevention; concerns about drug side effects; HIV stigma associated with pill-taking; negative reactions and lack of peer, family and/or partner support and ambivalence about taking a product of uncertain efficacy in a placebo-controlled clinical trial [56, 57]. In addition, data from FEM-PrEP study participants who acquired HIV infection indicate that women underestimated their risk and rationalized their risk behaviour, that perceived risk of HIV was associated with PrEP adherence [58], and that women perceived negative consequences of reporting non-adherence [59].

Notably, the qualitative research through the VOICE and FEM-PrEP trials did not indicate that women did not want biomedical HIV prevention, but that many encountered barriers to use and desired more directive feedback. Randomized trials are very different from real-world settings, as women are motivated to participate in trials for a variety of reasons (e.g. access to quality health services and monetary reimbursement for monthly visits). Study participants are counselled monthly that they may not be receiving active product, that the active product has uncertain efficacy, and they are provided with frequent reminders and encouragement to use product, which could have discouraged participants’ willingness to accurately report their adherence and study counsellors’ ability to address their concerns and barriers to adherence. Furthermore, study participants may feel less at risk over time in the context of monthly HIV testing, potentially coupled with less imminent concern about HIV in the trial communities, given expanded access to ART and declines in HIV mortality.

A part of the rationale for learning from implementation of oral PrEP for young African women is that it has shown efficacy in multiple populations, is already available globally in branded and lower cost generic formulations, and that other PrEP formulations (e.g. topical microbicides) have not yet consistently demonstrated efficacy. Specifically, 1% tenofovir gel dosed peri-coitally (two doses within 24 hours before and after sex) demonstrated moderate efficacy (39%) against HIV infection in the initial CAPRISA 004 trial in South Africa [60]. Disappointingly, no efficacy of tenofovir gel was observed in the confirmatory FACTS 001 trial of peri-coital dosing [61] or with daily dosing in the VOICE trial [52]; in both of those trials adherence to product was low. The results of FACTS 001 and VOICE may signal that vaginal gel may not be a practical or acceptable strategy for a large enough subset of women, as a result of privacy issues for storing and carrying applicators, vaginal wetness, leakage, dislike by male partners and the need to strategize about finding the right time for gel insertion; ongoing analyses from those trials will inform next steps, if any, for that delivery approach. Other microbicide approaches, for example, slow-release vaginal rings, are being evaluated in clinical trials.

### Prioritizing oral PrEP for young women

Uptake and adherence among participants in clinical trials who are randomized to placebo or active product and counselled about *unknown* efficacy may not predict uptake and adherence when patients in real-world settings are subsequently offered open-label product and counselled about *known* efficacy and the importance of adherence to achieve protection. Evidence of this has been demonstrated by the recent PROUD study, which offered immediate or deferred open-label daily PrEP among MSM in the UK and found high effectiveness (86% HIV protection) [62]. A demonstration project of PrEP, as a bridging strategy until the HIV infected partner was on ART for six months, was conducted among high-risk HIV serodiscordant couples in Kenya and Uganda, among whom HIV transmission was almost eliminated in this time-limited use of PrEP delivered with brief adherence counselling [63]. Notably, the majority of a cohort of single, young women from Cape Town, South Africa, took open-label oral PrEP when in the ADAPT/HPTN 067 study, in which women were randomized to one of three dosing schedules (daily, intermittent weekly with a “boost” at the time of sex, or coitally-dependent dosing). Daily dosing resulted in better coverage of sex acts and adherence, and higher drug levels [64]; daily dosing may foster better habit formation and provide the most forgiveness for missed doses.

These open-label PrEP studies demonstrate the feasibility of reaching at-risk populations and achieving higher effectiveness than was observed in placebo-controlled trials, in part due to populations who recognize their risk and are motivated to access a product with known high efficacy. These demonstration projects also indicate feasibility of public health delivery with quarterly visits, brief adherence counselling, and that adherence does not have to be perfect to achieve very substantial prevention benefits. The encouraging results from these initial PrEP implementation projects call for evaluation of open-label PrEP in young African women.

A key gap is whether when counselled about the high safety, tolerability and effectiveness of PrEP in other populations, young African women who have substantial risk of HIV will effectively use PrEP until more prevention options are available. Young women may have less agency and face different circumstances and barriers than men and motivated couples in using PrEP. Specific issues for young African women include stigma about being sexually active, and limited access to youth-friendly services and reproductive health services. Thus, implementation science research is critically needed on PrEP for young women to assess their motivations for HIV prevention, PrEP delivery models including through family planning clinics and with community partners, and adherence strategies to address barriers to young women's adherence to daily pill-taking with PrEP. Innovative strategies to mitigate these barriers may include those that draw from behavioural economics and treatment adherence research with adolescents and young people, as summarized below.

### Adopting new approaches for HIV prevention: behavioural economic and economic approaches

Behavioural economics, building on insights from psychology about predictable biases and mistakes that can make it hard for individuals to make healthy choices, provides a framework for understanding why young women find it hard to consistently engage in prevention even when they are informed about the benefits. “Present-bias” leads people to focus on immediate rewards or costs at the expense of their own long-term goals and objectives [65–67]. Health prevention decisions involve immediate costs (e.g. the annoyances and cost of engaging in prevention now) and delayed and uncertain rewards (e.g. avoiding HIV infection in the future). If young women are loss averse [68], or more concerned about losses than equivalent gains, they may respond strongly to the perception that their peers or sexual partners may stigmatize certain prevention decisions. In addition, many prevention strategies reduce but cannot eliminate risk, and messaging about partial efficacy can be confusing and ambiguous. Research indicates that adversity to ambiguity leads to inaction [69] and low levels of investment in risk reduction strategies [70].

During adolescence and their early 20s, many young women experience a period of rapid social, neurodevelopmental and physical growth. Studies indicate that brain development is not complete until the early 20s, with a lag between emotional and cognitive control maturation [71]. Neurocognitive development may affect integration of risk perception and behavioural decision-making by adolescents, and an evolving ability to weigh short-term intimacy and pleasure rewards versus longer term health promotion benefits [71, 72].

Even for young women fully intending to do everything they can to prevent HIV infection, prevention decisions are made on a continual basis. If adolescents have limited attention and are juggling chaotic lives with many priorities, threats and vulnerabilities such as poverty which reduces cognitive processing, prevention behaviours may suffer [73–75]. Additionally, if young women are not fully aware of their present-bias, tend to overly discount longer term benefits and are over-confident about their ability to adhere to prevention strategies, they may not spend enough time developing prevention strategies that can be consistently implemented [67]. Finally, “optimism bias” may be an important factor, where young women may tend to overestimate the risk of a behaviour (e.g. smoking or unprotected sex) for the health of others, but underestimate their own risks [76].

One strategy for overcoming present-bias against prevention would be to increase the rewards for prevention and bring them closer to the present. Randomized trials have shown success in interventions that financially incentivize individuals to engage in prevention behaviours such as medication adherence, weight loss and smoking cessation [77, 78], although the effects often wane after incentives are discontinued [78, 79]. Incentives in conjunction with peer mentoring have been shown to be effective for achieving glucose control in diabetics [80]. Because youth are particularly sensitive to peer influence, incentives with peer mentoring or modest group level incentives may be an effective approach in young women to support habit formation of regular adherence behaviours, such as with PrEP, but have not yet been evaluated.

The evidence on cash transfers and HIV prevention suggests that they may be useful tools for addressing structural barriers that increase young women's vulnerability to HIV, especially when provided at critical times. Given findings that women who have more education generally have lower HIV risk, interventions that keep adolescents in school would increase education levels in girls and potentially reduce their engagement in transactional sex [81]. A successful example of this strategy is an evaluation of conditional cash transfers for girls’ secondary school attendance in Malawi and unconditional cash transfers, both of which showed lower HIV and HSV-2 prevalence and positive changes in the age of young women's sex partners and frequency of sex acts [82]. Other trials of conditional cash transfers to incentivize school attendance have been conducted with HIV incidence endpoints, including a trial in South Africa, which will have results in the next year [83]. Unconditional cash transfers have been associated with delayed onset of sexual activity among orphans and vulnerable children in Kenya [84], and reduced prevalence of transactional sex and age-disparate sex for adolescent girls in South Africa [85]. Cash transfers may enable women to select different partners to avoid HIV risk or they may provide bargaining power for women in relationships that are unequal. Evidence for incentives that are conditioned specifically on the outcome of staying STI and HIV negative is mixed; women have less risky sex but men receiving incentives engaged in more risky behaviour [86]. Other studies have found evidence of a reduction in bacterial STIs after one year of incentive payments [87], and a reduction in in HIV incidence through a lottery incentive scheme [88].

More work is needed to understand the mechanism driving divergent results on incentives for HIV avoidance, and for attaining sustained responses after incentives end. Although the psychology literature does not suggest that incentives “crowd out” intrinsic motivation when the baseline motivation for behaviours is low [89], more understanding is needed about whether incentives alter individuals’ intrinsic motivation to do things that are good for their health as well as the feasibility, sustainability and cost-effectiveness of incentive programs.

### Supporting adherence to oral PrEP for young women

PrEP is not suitable for all persons at risk, but may be feasible for a substantial portion of persons during “seasons” of high risk [90], which may differ by population and setting. Analysis of drug levels in the oral PrEP efficacy trials demonstrate that the majority of participants self-sorted within the first three months into users versus non-users [50]. One goal of PrEP implementation projects is to understand the motivations of persons at risk of HIV to use PrEP and to help support formation and continuation of early adherence behaviours. For young women who have had limited healthcare utilization and minimal experience with long-term medications, PrEP adherence support should focus on practical issues, including counselling about possible early and transient gastrointestinal symptoms, dosing, product storage, “pocket doses” when away from home, and incorporation of a regular behaviour like pill-taking with daily routines. Peer support and modest, short-term incentives might provide additional support for adherence to PrEP, but need to be evaluated. Given the importance of peers and social norms among young women, incentives for PrEP adherence may be more powerful if delivered as part of peer adherence mentoring and support.

Young women may have greater difficulty with adherence than older women and other populations, in part due to cognitive development [91]. Psychological and social barriers are significant predictors of non-adherence among studies of adolescents with a range of chronic illnesses with medication adherence rates ranging from 20 to 60% [92–95]. Poor adherence to ART has been linked to depression, gender-based violence and lack of social support, all of which are common in young people in sub-Saharan Africa [96–99]. Lower rates of adherence to preventive health interventions have been observed among youth, as demonstrated by lower adherence to oral contraceptives among adolescent and young adult women at high risk of unintended pregnancy [100–102].

With respect to adherence support for young African women, recent meta-analyses highlight that cognitive-behavioural problem-solving [103, 104] and peer support [105] consistently improve medication adherence among adults in the United States and developing countries. Cognitive-behavioural approaches have been used to treat youth depression [106] and trauma [107], improve adult and youth ART adherence [104, 105, 108] and decrease alcohol use in HIV-infected sub-Saharan African adults [109]. Cognitive-behavioural therapy (CBT) is a brief, cost-effective intervention strategy that can be implemented by a wide range of providers, including non-specialists and those with little counselling experience, in both clinical and community-based settings in low resource settings [110–112]. Most research on adherence counselling based on CBT is from the treatment field, and evaluation is needed on CBT to support prevention behaviours. The Life Steps programme is an example of CBT, incorporating informational, problem-solving and cognitive-behavioural strategies to address barriers to ART adherence, which was successfully used to support PrEP adherence among HIV serodiscordant couples in the Partners PrEP Study [113], and could be adapted to counsel young women about PrEP adherence challenges.

The revolution in mobile phone technology creates new opportunities to engage youth and build new social relationships and networks with adolescents. These opportunities can be leveraged for health. SMS reminders provide cognitive reminders for adherence and can strengthen communications between patients and providers [114]. A recent meta-analysis found that text messaging can be an effective support for ART adherence, particularly with messaging provided on a less than daily basis, content and timing that is individually tailored and platforms designed to evoke a reply from the recipient [115]. SMS have also increased oral contraceptive continuation rates [116] and could be incorporated to provide cognitive reminders for PrEP among women as they are establishing adherence habits.

## Conclusions: priorities for implementation of oral PrEP for young African women

With strong evidence for the efficacy and effectiveness of oral PrEP across multiple studies, it is essential to understand whether young women will be interested in using PrEP and able to sustain adherence in the context of clear positive messaging and integrated with delivery of other services that meet young women's needs (e.g. family planning, emergency contraception, post-exposure prophylaxis, IPV and skills training). PrEP demonstration projects for young African women provide an opportunity for evaluation of delivery models, innovative communications, adherence and behavioural economics interventions to support uptake of effective, novel HIV prevention interventions. These opportunities include evaluating strategies to make risk salient to young women, understand and maximize women's motivations for prevention, and use of social support and social media to support PrEP uptake adherence, and delivery models, as summarized in Table 1.

**Table 1:** Considerations for future research with oral PrEP for young African women

Topic	Strategy
1. Understand the end-users for HIV prevention during product development and delivery	• Conduct formative research to understand young African women's needs and preferences about products and delivery strategies for HIV prevention. Methods that could be useful include ethnographic research as part of behaviour-centred and user-centred design, and mental models approaches • Evaluate young women's decision-making about HIV prevention • Assess end-users’ preferences in designing new products
2. Test communication messages and demand creation strategies	• Rigorously test different communication messages to determine which are most salient and young women respond to most strongly, including: ○ Emotion and desire for status are important drivers of decisions ○ Deemphasize messages about risk, which is ambiguous, dynamic and can stigmatize sexuality, potentially increasing acceptability for using HIV prevention methods ○ Frame prevention messaging positively in terms of benefits in intimacy, self-esteem and desire to achieve one's aspirations ○ Identify ‘positive deviants’ and disseminate compelling narratives from women who have successfully utilized PrEP • Identify effective strategies for demand creation, including ones from implementation of contraceptive services. • Use technology to achieve “wide reach” in disseminating messages and promoting HIV prevention, with interactive platforms, such as social media
3. Develop innovative interventions to motivate HIV prevention behaviours, including PrEP	• Use formative research informed by behaviour-centred design to identify evolutionary motivators and pilot interventions addressing these motivators • Identify ‘levers’ for initiation of PrEP and continued use, pilot and evaluate interventions to increase these behaviours • Identify ways to make pill-taking behaviours automatic and part of young women's routine practices • Evaluate whether a validated empiric risk score increases the uptake of oral PrEP among young women with a significant risk of HIV acquisition • Address medication maintenance factors such as transport and financial barriers for refills, and stigma
4. Evaluate PrEP effectiveness among young African women	• Conduct a demonstration project of open-label oral PrEP among young African women with HIV incidence as the primary outcome, either using a counterfactual with HIV rates in recent trials among women of similar risk or in an immediate-deferred design (such as the PROUD study among MSM in the UK)
5. Test delivery models of PrEP, including integration with family planning and other services	• Evaluate whether uptake of HIV prevention is higher if offered with contraceptive counselling and services (e.g. cervical cancer and STI screening, gender-based violence counselling) as a separate service, and through clinics or community programmes • Test different HIV prevention delivery models, including offering PrEP when women seek contraception (e.g. injectables, implants) or emergency contraception and post-exposure prophylaxis. • As additional PrEP formulations become available (e.g. vaginal rings, injectables), evaluate the family planning delivery model of providing women with choice of methods • Assess cost-effectiveness of delivery of PrEP in demonstration projects
6. Assess behavioural economic approaches to HIV prevention	• Assess whether group or individual incentives are effective for young women's initiation of and/or adherence to PrEP. • Evaluate HIV prevention technologies with measurement of the barriers between inaction and action (e.g. present bias, limited attention, cognitive capacity, bounded rationality including partial information, rumour, inaccuracies about sex and HIV) • Deliver HIV prevention with a focus on convenience, bundling with other services, simple and clear messages about benefits/costs and reminders

The effectiveness of positive communication messages designed using insights from ethnographic research, mental models [117] and other behavioural models should be evaluated to determine whether effectively framed messages about how PrEP helps young women meet their aspirations, and achieve greater intimacy, self-esteem, love and confidence resonate with young women more than messages that are framed with disease prevention as the only benefit [118]. Behavioural economics could be useful in smarter design of communication campaigns about PrEP, by incorporating and addressing biases in decision-making. Furthermore, insights from behavioural economics could be useful in focusing attention on beliefs and emotions in making decisions [119]. Evaluation is needed of social marketing of HIV prevention including PrEP, using the internet for promotion of effective HIV interventions, as well as digital games, phone apps and narratives to provide more salient and effective methods for engaging youth and correcting incorrect beliefs and perceptions about HIV risk [120]. Demonstration projects should assess the use of risk scores that stratify women according to their risk of HIV; one example is the risk score developed from analyses of HIV seroconverters in the VOICE trial [121].

PrEP delivery models need to be piloted for young women who have limited access to reproductive and sexual health services in many parts of Africa, in part due to provider attitudes towards sexually active unmarried women. Family planning clinics could be an efficient, existing delivery site for PrEP, by reaching women with overlapping risks for pregnancy and HIV and creating synergies in integrated rather than parallel delivery programmes. Women who are seeking services at youth-friendly clinics or family planning services are potential early adopters of PrEP because they are already engaging with the healthcare system and are already motivated by prevention (i.e. to receive reproductive health services and to reduce their risk of pregnancy). In addition, in some African countries legislation is needed to permit adolescents to access sexual and reproductive health services to remove this structural barrier to therapeutic and prevention services in general, including PrEP.

PrEP implementation studies for young African women need to evaluate sociocultural contextual issues, caregiver support, peer norms and relations, and cognitive and environmental factors that influence uptake and adherence to biomedical HIV preventions. Ethnographic research is needed to better understand sexual partnerships, social influencers, and household factors (e.g. composition, stability, privacy and communication about sexuality) that shape young women's agency, priorities and ability to seek and adopt HIV prevention, including PrEP. Given the importance of context, sexual norms and expectations, and structural factors on increasing risk and vulnerability for young African women, PrEP may have greater uptake and adherence if it is provided in combination with counselling, peer support and referrals for women who have experienced sexual violence, have alcohol dependence or other needs^122^. PrEP demonstration projects are being launched for sex worker populations in Africa to understand delivery, uptake and adherence to PrEP, with the assumption that sex workers may have different motivations, barriers and delivery strategies for PrEP than young African women in the general population.

PrEP demand should be quantified in terms of uptake and continuation rates among women of different ages and partnership characteristics in different settings as part of market segmentation research, which could also help forecast programme costs and supply chain needs. Scalable adherence strategies, such as brief counselling based on CBT, two-way interactive SMS messages as cognitive reminders, peer support groups and short-term incentives to support adherence behaviours need to be evaluated among young African women in open-label PrEP demonstration projects. Demonstration projects will be most policy informative if they are sufficiently large and designed to assess HIV incidence, either through a counterfactual or with immediate versus delayed PrEP use.

Oral PrEP is a first-generation product, which has many similarities to the oral contraceptive pill which also has adherence challenges but made a significant impact on pregnancy prevention and paved the way for a multitude of contraceptive methods [123]. Adherence to daily pill-taking may limit the market segment of women who are able to benefit from PrEP, but this needs to be assessed and quantified. However, important lessons can be learned from demonstration projects of PrEP, as the demonstration projects among MSM in Europe and couples in Africa have demonstrated, which showed that men and couples at high risk for HIV were able to take PrEP with sufficient adherence to achieve a very substantial HIV prevention benefit. Although there is great enthusiasm for the potential of longer-acting products to address adherence challenges of daily pill-taking with PrEP, these may also be associated with issues about uptake, if use of any formulation of PrEP requires risk perception and engagement with HIV prevention to generate sufficient interest. Lessons learned through implementation of oral PrEP, a first-generation biomedical HIV prevention product, will inform development of new PrEP formulations, including intravaginal rings and injectable PrEP, with the goal to eventually offer an expanding choice of prevention options in integrated, combination programmes for young African women.
